# Supplementary material for: Biological rhythms in the deep-sea hydrothermal mussel Bathymodiolus azoricus
Source: Nat Commun. 2020 Jul 10;11:3454. doi: 10.1038/s41467-020-17284-4 (PMC7351958; doi:10.1038/s41467-020-17284-4)
Supplement: Supplementary file 1 — Supplementary Information [file 41467_2020_17284_MOESM1_ESM.pdf]

Supplementary Information

Biological rhythms in the deep-sea hydrothermal mussel *Bathymodiolus azoricus*

Mat *et al*.

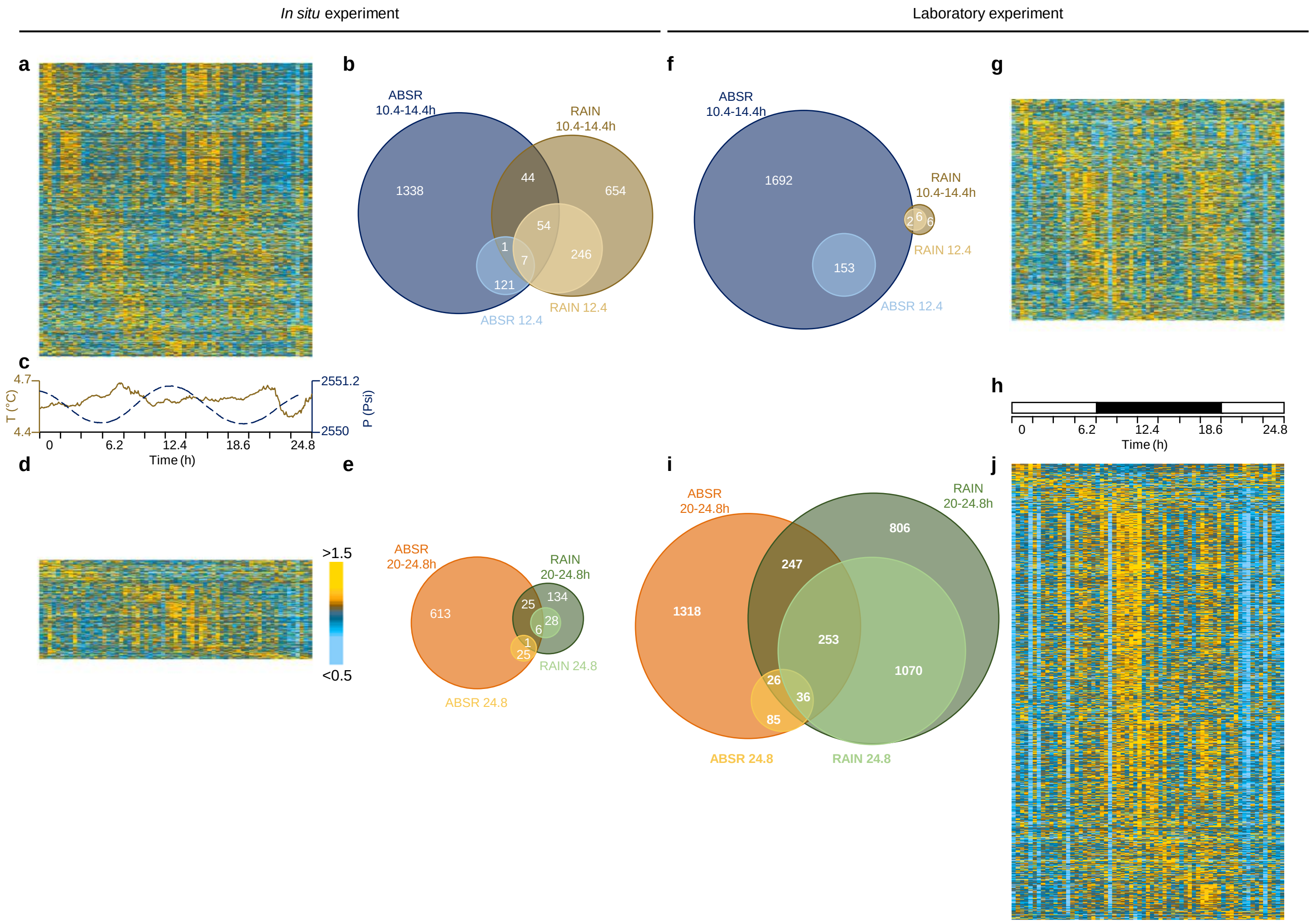

k

	In situ experiment		Laboratory experiment	
	Median cpm	Annotation (%)	Median cpm	Annotation (%)
Reference	7.0	47.2	5.3	47.2
Tidal transcripts (10,4-14,4h)	7.2	44.7	6.7	40.7
Daily transcripts (20-24,48h)	7.0	48.6	5.5	57.6

Supplementary Figure 1. *Bathymodiolus azoricus* gill transcriptome is cyclic both at the hydrothermal vent field and in the laboratory. Normalisation: down-sampling. a-e, *In situ* experiment. **a,** Median-normalised expression patterns of all 2,465 rhythmic transcripts detected in the interval 10.4-14.4 h and presented as a heatmap. **b**, Euler diagrams detailing the number of rhythmic transcripts detected in the interval 10.4-14.4 h and specifically with a period of 12.4 h using both RAIN (dark and light brown disks) and ABSR (dark and light blue disks) methods. **c**, Seawater temperature (brown continuous line) and pressure (blue dotted line) profiles recorded on the LS vent field at the time of sampling. **d,** Median-normalised expression patterns of all 832 rhythmic transcripts detected in the interval 20-24.8 h and presented as a heatmap. **e**, Euler diagrams showing the number of rhythmic transcripts detected in the interval 20-24.8 h and specifically with a period of 24.8 h using both RAIN (dark and light green disks) and ABSR (dark and light orange disks) methods. **f-j,** Laboratory experiment. **f,** Euler diagrams detailing the number of rhythmic transcripts detected in the interval 10.4-14.4 h and specifically with a period of 12.4 h using both RAIN and ABSR methods. **g**, Median-normalised expression patterns of all 1,859 rhythmic transcripts detected in the interval 10.4-14.4 h and presented as a heatmap. **h**, Light:Dark 12h:12h cycle implemented in the laboratory. **i,** Euler diagrams showing the number of rhythmic transcripts detected in the interval 20-24.8 h and specifically with a period of 24.8 h using both RAIN and ABSR methods. **j**, Median-normalised expression patterns of all 3,841 rhythmic transcripts detected in the interval 20-24.8 h and presented as a heatmap. **k**, Comparison of the subsamples of rhythmic transcripts with the reference transcriptome in terms of counts (cpm: counts per million reads) and percentage of annotated transcripts.

All heatmaps are single-plotted and represent 5 individuals per time point. Rhythmic transcripts are ordered by phase. Heatmap colours: median-normalised expression levels >1.5-fold are shown as gold yellow; expression levels <0.5-fold are shown as light blue. Heatmap heights and disk areas are proportional to the number of transcripts.

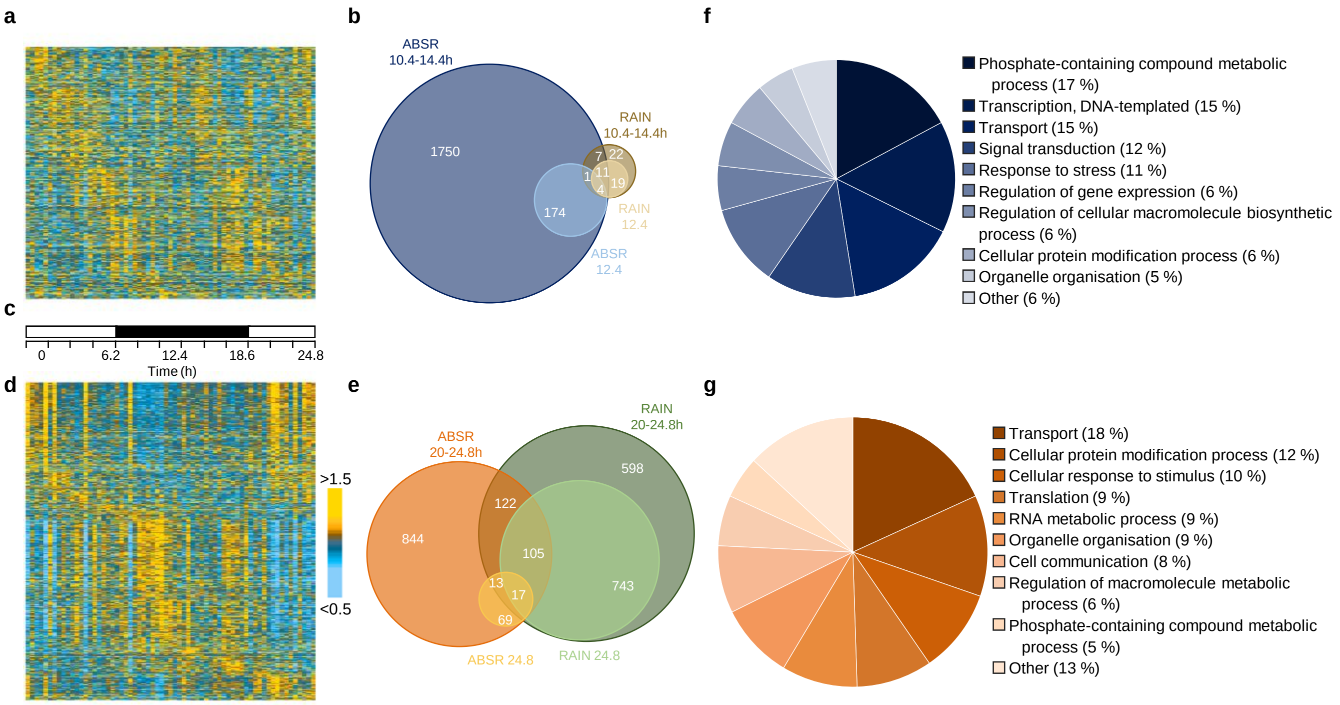

Supplementary Figure 2. *Bathymodiolus azoricus* gill transcriptome was cyclic in the laboratory under L:D 12h:12h. Normalisation performed with down-sampling and DESeq2. a, Heatmap of median-normalised expression patterns of all 1,988 rhythmic transcripts detected in the interval 10.4-14.4 h. **b,** Euler diagrams detailing the number of rhythmic transcripts detected in the interval 10.4-14.4 h and specifically with a period of 12.4 h using both RAIN (dark and light brown disks) and ABSR (dark and light blue disks) methods. **c,** Light:Dark 12h:12h cycle implemented in the laboratory. **d,** Heatmap of median-normalised expression patterns of all 2,511 rhythmic transcripts detected in the interval 20-24.8 h. **e,** Euler diagrams showing the number of rhythmic transcripts detected in the interval 20-24.8 h and specifically with a period of 24.8 h using both RAIN (dark and light green disks) and ABSR (dark and light orange disks) methods. **f,** GO terms associated to all the rhythmic transcripts detected in the range 10.4-14.4 h and **g,** in the range 20-24.8 h.

All heatmaps are single-plotted and represent 5 individuals per time point. Rhythmic transcripts are ordered by phase. Heatmap colours: median-normalised expression levels greater than 1.5-fold are shown as gold yellow; expression levels less than 0.5-fold are shown as light blue. Heatmap heights and disk areas are proportional to the number of transcripts.

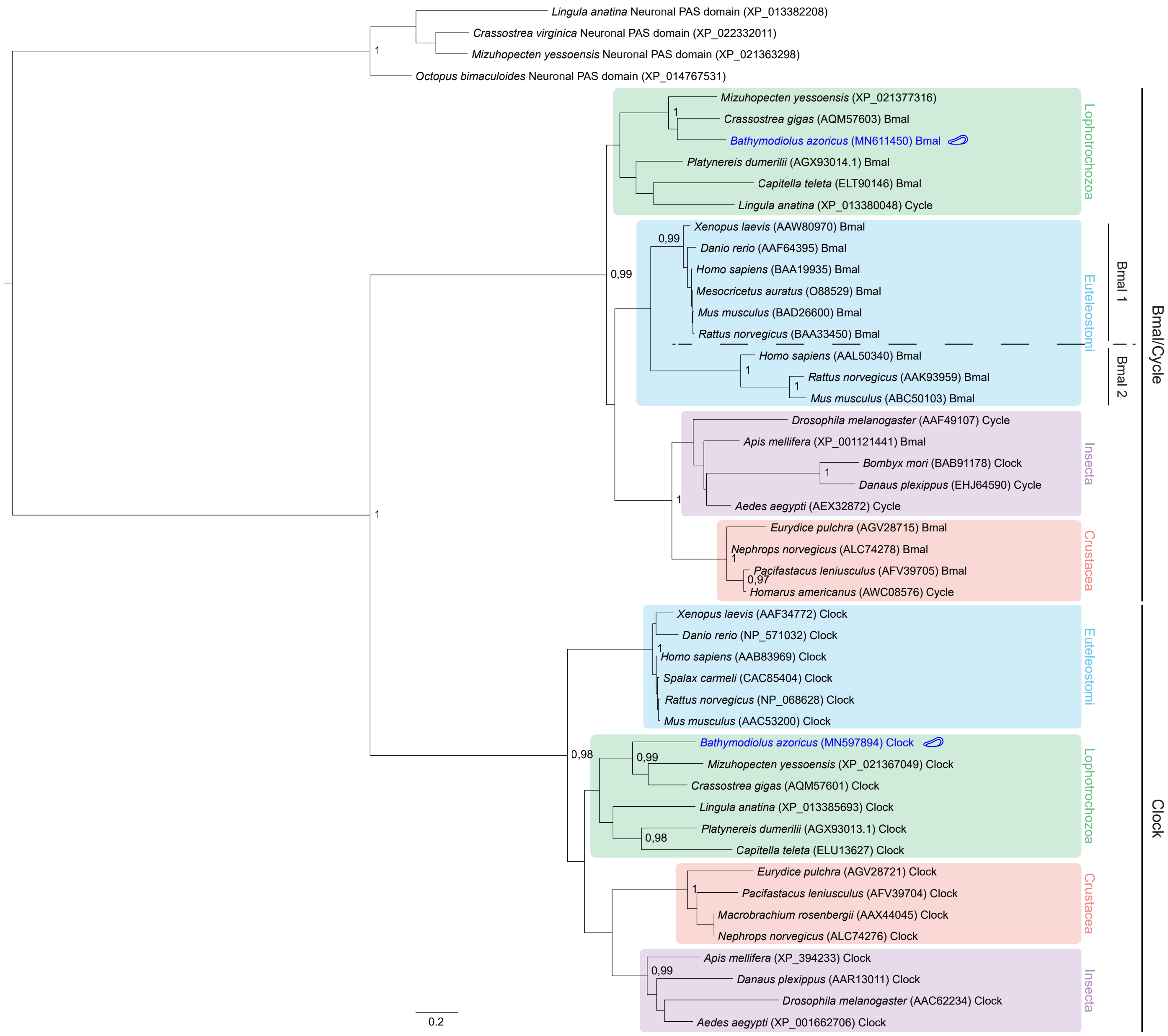

Supplementary Figure 3. Maximum-likelihood phylogeny of the CLOCK and BMAL/CYCLE proteins. Nodes supported by an aLRT value >=0.97 are indicated. *Bathymodiolus azoricus* proteins are highlighted in blue.

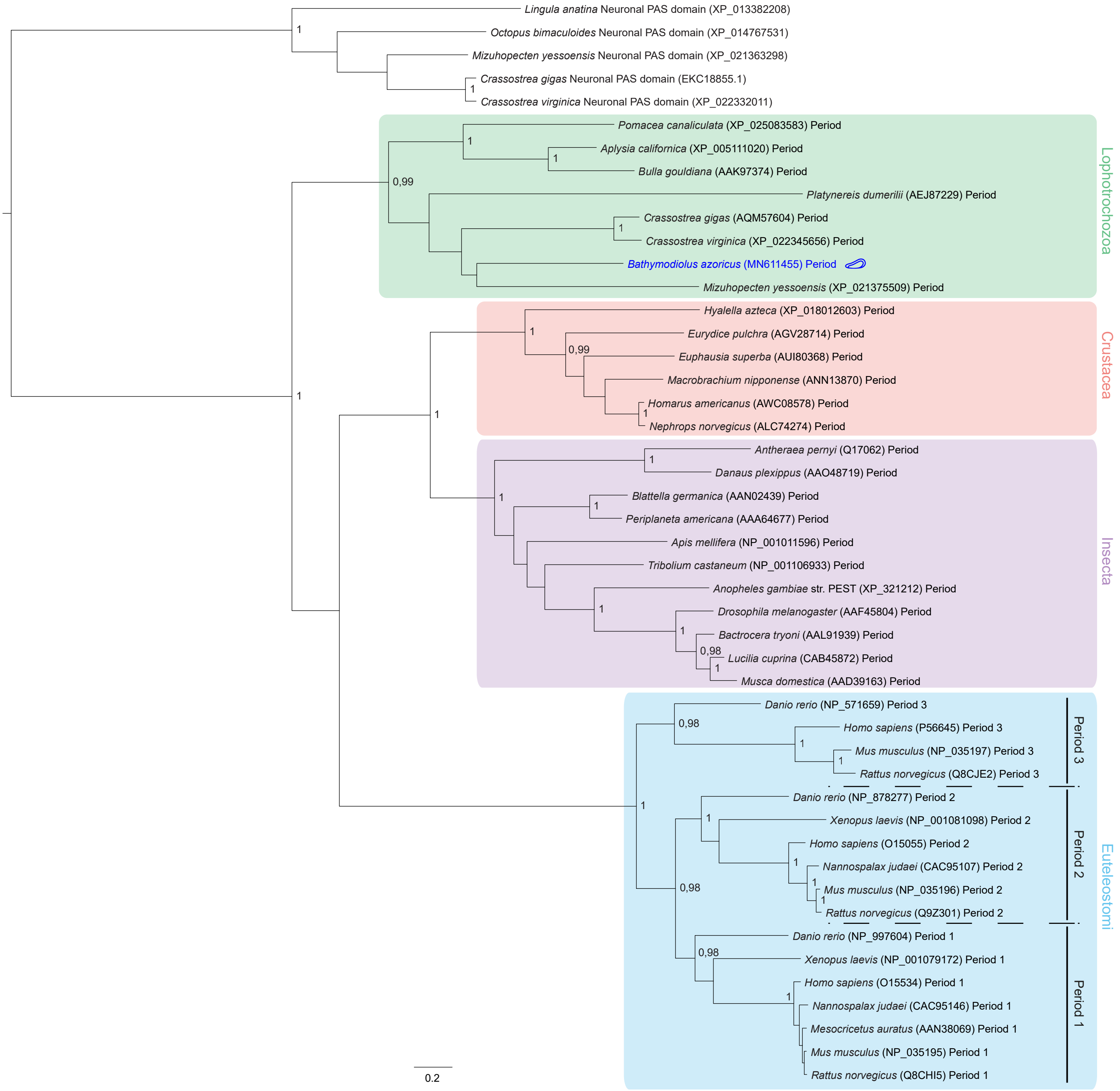

Supplementary Figure 4. Maximum-likelihood phylogeny of the PERIOD proteins. Nodes supported by an aLRT value >=0.97 are indicated. *Bathymodiolus azoricus* proteins are highlighted in blue.

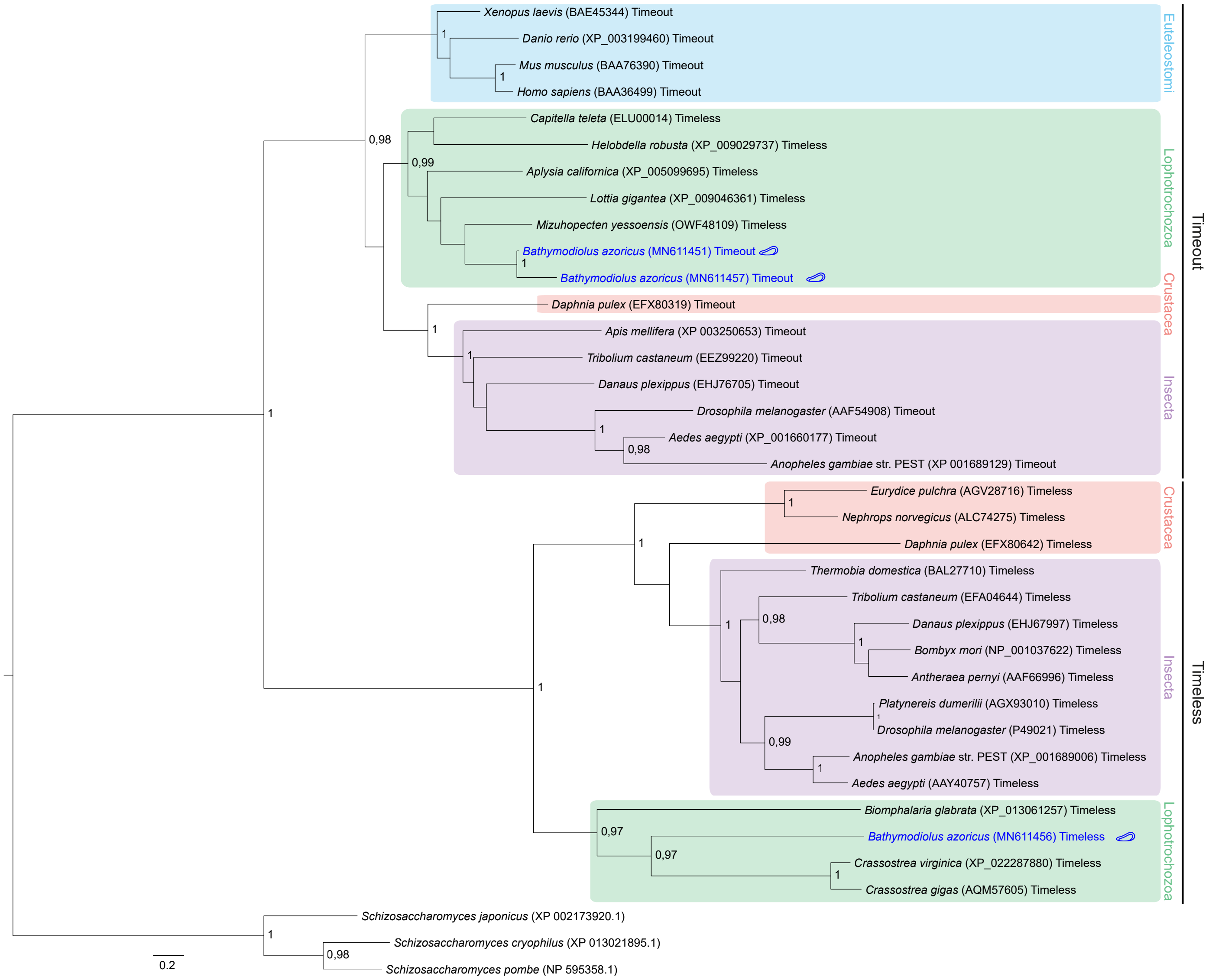

Supplementary Figure 5. Maximum-likelihood phylogeny of the TIMELESS and TIMEOUT proteins. Nodes supported by an aLRT value >=0.97 are indicated. *Bathymodiolus azoricus* proteins are highlighted in blue.

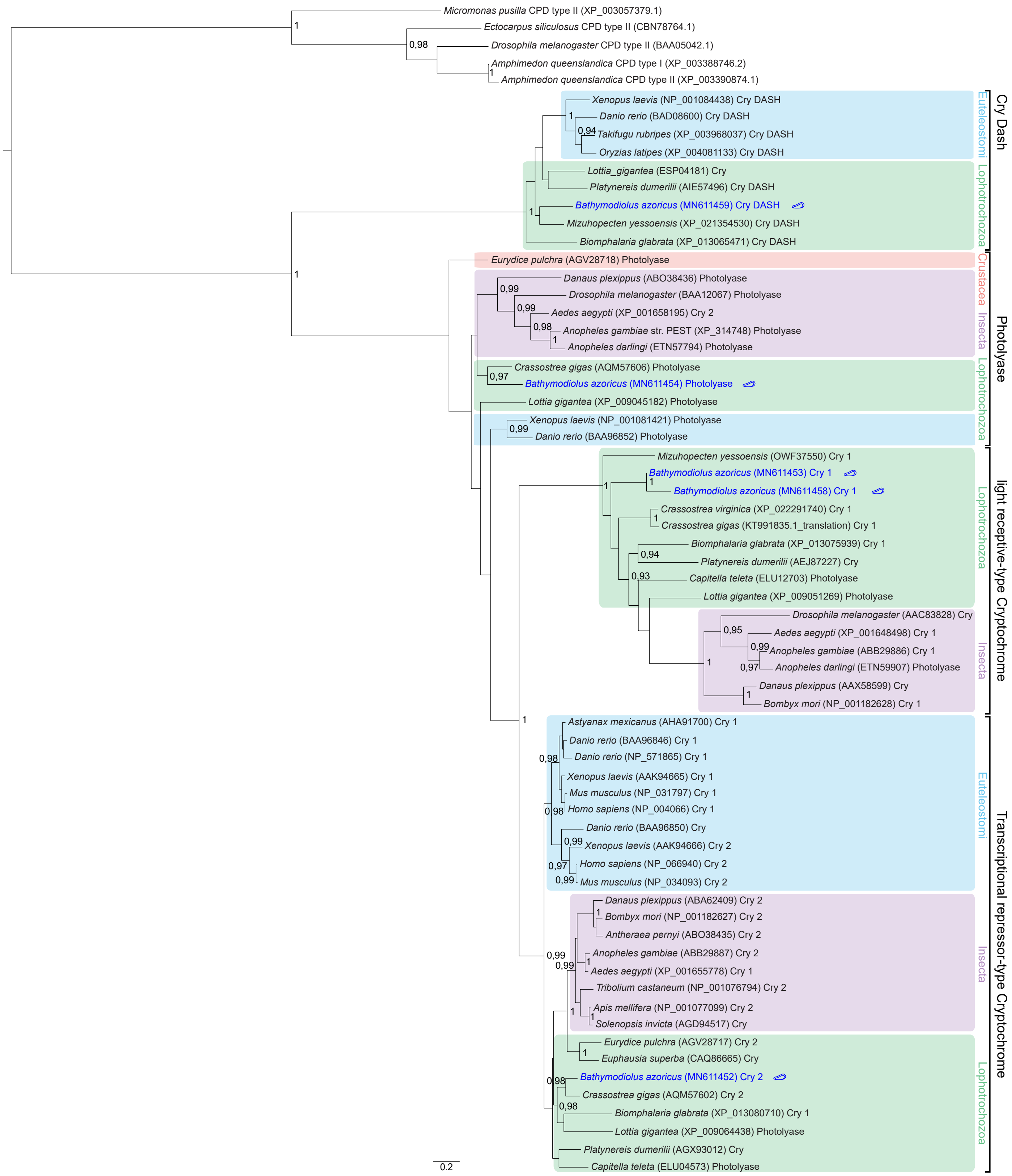

Supplementary Figure 6. Maximum-likelihood phylogenies of the CRYPTOCHROME and PHOTOLYASE proteins. Nodes supported by an aLRT value >=0.97 are indicated. *Bathymodiolus azoricus* proteins are highlighted in blue.

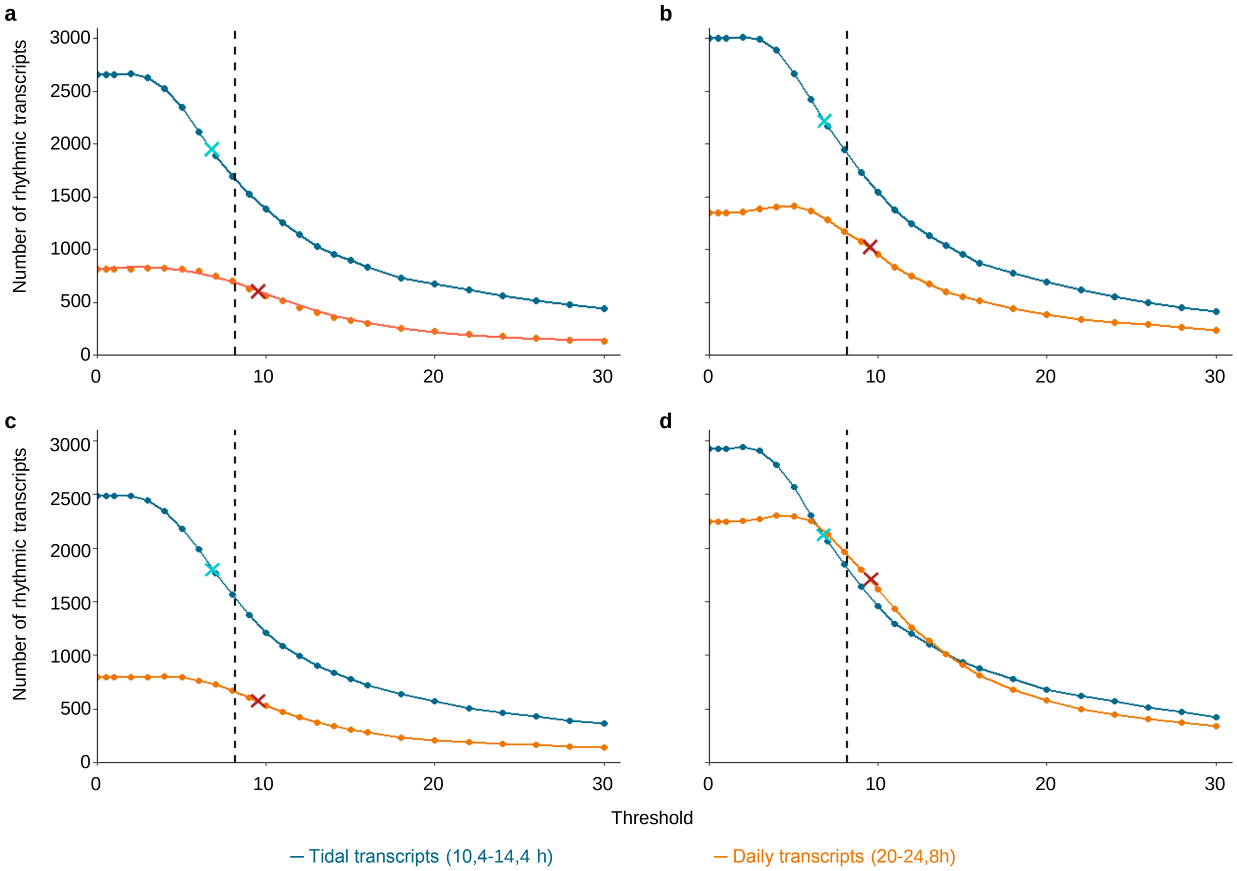

Supplementary Figure 7. Threshold setting for the ABSR method. a-b, Data normalised using both down-sampling and DESeq2. **a,** Spectrum thresholds from 0 to 30 for the *in situ* experiment. **b,** Spectrum thresholds from 0 to 30 for the laboratory experiment. **c-d,** Data normalised with down-sampling. **c,** Spectrum thresholds from 0 to 30 for the *in situ* experiment. **d,** Spectrum thresholds from 0 to 30 for the laboratory experiment.

Top blue curve: number of tidal transcripts. Bottom orange curve: number of daily transcripts. X: inflection points for each curve. Vertical dotted line: mean of the two inflection points.
