# Supplementary material for: Biological rhythms in the deep-sea hydrothermal mussel Bathymodiolus azoricus
Source: Nat Commun. 2020 Jul 10;11:3454. doi: 10.1038/s41467-020-17284-4 (PMC7351958; doi:10.1038/s41467-020-17284-4)
Supplement: Supplementary file 3 — Reporting Summary [file 41467_2020_17284_MOESM3_ESM.pdf]

nature research

Corresponding author(s): Audrey M Mat and Marjolaine Matabos

Last updated by author(s): Jun 15, 2020

Reporting Summary

Nature Research wishes to improve the reproducibility of the work that we publish. This form provides structure for consistency and transparency in reporting. For further information on Nature Research policies, see Authors & Referees and the Editorial Policy Checklist .

Statistics

For all statistical analyses, confirm that the following items are present in the figure legend, table legend, main text, or Methods section.

n/a	Confirmed	
☐	☒	The exact sample size (*n*) for each experimental group/condition, given as a discrete number and unit of measurement
☐	☒	A statement on whether measurements were taken from distinct samples or whether the same sample was measured repeatedly
☐	☒	The statistical test(s) used AND whether they are one- or two-sided<br>*Only common tests should be described solely by name; describe more complex techniques in the Methods section.*
☐	☒	A description of all covariates tested
☐	☒	A description of any assumptions or corrections, such as tests of normality and adjustment for multiple comparisons
☐	☒	A full description of the statistical parameters including central tendency (e.g. means) or other basic estimates (e.g. regression coefficient) AND variation (e.g. standard deviation) or associated estimates of uncertainty (e.g. confidence intervals)
☐	☒	For null hypothesis testing, the test statistic (e.g. *F*, *t*, *r*) with confidence intervals, effect sizes, degrees of freedom and *P* value noted<br>*Give P values as exact values whenever suitable.*
☒	☐	For Bayesian analysis, information on the choice of priors and Markov chain Monte Carlo settings
☒	☐	For hierarchical and complex designs, identification of the appropriate level for tests and full reporting of outcomes
☒	☐	Estimates of effect sizes (e.g. Cohen's *d*, Pearson's *r*), indicating how they were calculated

Our web collection on statistics for biologists contains articles on many of the points above.

Software and code

Policy information about availability of computer code

Data collection	No software was used for data collection
Data analysis	Softwares or packages used to analyse the data are: R (v 3.5.0), the R-package adespatial (v 0.3-7), fastQC (https://www.bioinformatics.babraham.ac.uk/projects/fastqc/ version 0.11.2), DRAP (version 1.91), the BWA-MEM algorithm (version 0.7.12-r1039), BUSCO (v3), TransDecoder (version 3.0.0), Interproscan (v5), diamond (version v0.9.9), the KEGG database (june 2018), SAMtools (version 1.3.1), the R-package DESeq2 (v 1.22.2), the R-package RAIN (v 1.16.0), the R code ABSR (2018), the R-package pheatmap (v 1.0.12), Blast2GO (v.5.2.5), the R-package topGO (v.2.34.0), MAFFT (v7.388), IQtree (v1.6.7), PhyML (v3.3.2).

For manuscripts utilizing custom algorithms or software that are central to the research but not yet described in published literature, software must be made available to editors/reviewers. We strongly encourage code deposition in a community repository (e.g. GitHub). See the Nature Research guidelines for submitting code & software for further information.

Data

Policy information about availability of data

All manuscripts must include a data availability statement. This statement should provide the following information, where applicable:

- Accession codes, unique identifiers, or web links for publicly available datasets
- A list of figures that have associated raw data
- A description of any restrictions on data availability

Metadata from the Momarsat 2017 cruise are available under the doi [doi.org/10.17600/17000500]. RNA-seq data were deposited in the ArrayExpress database at EMBL-EBI [www.ebi.ac.uk/arrayexpress] under accession number E-MTAB-8451. The ENA study accession is ERP117902. The data and de novo reference transcriptome are also in the SEXTANT database under the doi [doi.org/10.12770/971d2c1a-51cc-49fd-882c-465970de8ed2]. Circadian clock genes sequences were deposited in Genbank under the following accession: bmal, MN611450; Clock, MN597894; period, MN611455; timeless, MN611456; timeout1, MN611457; timeout2, MN611451; cry1a, MN611458; cry1b, MN611453; cry2, MN611452; 6-4 photolyase, MN611454; cry DASH, MN611459. Raw data associated with Fig 5 are provided in the Source Data file.

Field-specific reporting

Please select the one below that is the best fit for your research. If you are not sure, read the appropriate sections before making your selection.

☐ Life sciences ☐ Behavioural & social sciences ☒ Ecological, evolutionary & environmental sciences

For a reference copy of the document with all sections, see nature.com/documents/nr-reporting-summary-flat.pdf

Ecological, evolutionary & environmental sciences study design

All studies must disclose on these points even when the disclosure is negative.

Study description

In the present study, we provided behavioural data for the deep-sea vent mussel Bathymodiolus azoricus, and sequenced high-resolution temporal transcriptomes of B. azoricus both in situ on the Mid-Atlantic Ridge (MAR), and in the laboratory.

Research sample

The organism studied is the deep-sea Mytilidae Bathymodiolus azoricus.
Behavioural data were recorded at the base of the Eiffel Tower edifice (Lucky Strike vent field, MAR).
The in situ transcriptome was performed on mussels from the Lucky Strike vent field on the Eiffel Tower edifice (1,688 m depth), which is below the 1,000 m depth limit of the twilight zone and thus blind to L:D cycle. The sampling location was chosen because the site was similar as the one for behavioural studies, and was protected from the white lights of the ROV operating in previous dives.
The laboratory transcriptome was performed on mussels from the nearby Menez Gwen vent field (834 m depth) that can be studied at atmospheric pressure.
Population: not applicable.
The sex of the animals could not be determined. The age of deep-sea mussels is currently an open question in deep-sea biology. As a proxy, the size of the animals is provided: 67 ± 13 mm shell length (mean ± sd, n=65 animals) for the in situ experiment; 63 ± 8 mm shell length (mean ± sd, n=65 animals) for the laboratory experiment.

Sampling strategy

No statistical method was used to predetermine sample size because this was a fully exploratory study performed in a non-model organism whose variability was unknown. Sample size was chosen as a compromise between deep-sea technical constraints, the number of replicates, and the sequencing effort which determines the cost.

Deep-sea technical constraints and number of replicates: the sampling box used to fix samples in situ had a volume of ~5L and samples had to be preserved in a 5-10 times volume of stabilising solution. As mussels have a mean volume of ~50 mL (without the shell), this allowed to sample 10-20 mussels, theoretically. It is recommended to conduct responsible science at hydrothermal vents and to preserve deep-sea ressources (Devey et al., 2007, Responsible science at hydrothermal vents. Oceanography 20(1):162–171), so we aimed at a reduced sample size. Additionally, it is not possible to take a pre-defined number of mussels using the arm of the ROV, so the exact number of mussels collected is determined once the samples are back on board. During samples collection, mussels were intentionally slightly cracked open using the ROV arm, and directly placed in a box to preserve RNA immediately upon sampling. Once on board, mussels with unbroken shells were discarded.

Sequencing effort and costs: based on the samples we had been able to collect, and on the authors' expertise in marine bivalve and sequencing strategies, we opted for n=5 for each time point. Based on our expertise, n=5 is a rather high number of replicates; and was chosen because the variability in gene expression was unknown in this context and species. Also, we wanted to sequence those replicates with the latest sequencing techniques available. Indeed, the detection of rhythmic transcripts depends on read depth (see Discussion). For marine emerging model organisms including Molluscs, for which the genome is not available, at least 20 million reads are required for tissue samples intended for de novo transcriptome assembly (Francis et al., 2013). Furthermore, 10-20 and 20-40 million reads have been recommended to detect 75-100 % of circadian transcripts in flies and mice respectively (Li et al., 2015).

After the sequencing, we used our data to confirm that our sampling strategy was consistent. This was first indicated by the results we obtained. We also used scotty (http://scotty.genetics.utah.edu/ ; Busby et al. 2013, Bioinformatics 1;29(5):656-7) to assess our experimental design. Based on our data, it showed that the least expensive and robust experimental design was to use 3 to 4 replicates sequenced to a depth of 10 million reads. The sample size was thus sufficient for the study. And the sequencing provided 92.0 ± 29.3 and 93.2 ± 16.3 millions of read pairs per library for the in situ and laboratory experiments, respectively. So, our sampling strategy was consistent.

Behavioural data were recorded with the ecological observation module TEMPO, which is part of the EMSO-Azores observatory and allows the direct observation of a B. azoricus mussel assemblage. We observed all mussels that could be tracked over the entire study period, corresponding to 31 mussels out of 90.

For the in situ transcriptome, mussels were collected (MOMARSAT 2017 cruise) and immediately stabilised under red light on the NE part of the Eiffel Tower edifice, at a depth of 1,688 m, using the Remotely-Operated Vehicle (ROV) Victor6000. Mussels were sampled every 2h 4min for 24h 48min. For the laboratory experiment, mussels were sampled during the Biobaz 2017 cruise at the Menez Gwen vent field of the Mid-Atlantic Ridge, White Flames area, at a mean depth of 834 m, using the ROV Victor6000.

Data collection

Behavioural data were gathered and analysed by MM:
Behavioural data were recorded with the ecological observation module TEMPO, located at the base of the Eiffel Tower edifice (LS vent field, MAR). The data analysed in this study were collected from July 27, 2014 to August 31, 2014, during which the camera recorded 2-min video sequences four times a day. Thirty one mussels could be tracked over the entire period. For each video, a snapshot extracted at the 25th second was analysed to determine if each individual mussel was open (coded as 1) or closed (coded

by 0) using Image J©. The proportion of opened mussels was then calculated for each observation date and the resulting time-series was submitted to a periodogram analysis using the R-package adespatial.

Environmental data:
Temperature and pressure data were recorded every 2 min by a SBE 53 Bottom Pressure Recorder (Sea-bird Scientific) 26 s/n located on the LS vent field at a depth of 1,726 m, and located at a distance of 745 m from our sampling site.

Transcriptomic data:
RNA were extracted from gill tissues by AMM. Gills tissues from both the in situ and lab experiments were multiplexed on one S4 flow cell lane, and paired-end lengths of 2x150 bp were sequenced on an Illumina NovaSeq system (Illumina, California, USA) at the GeT-Plage core facility (Toulouse, France; http://www.get.genotoul.fr) by CE. Bioinformatic analyses were performed by CK and AMM. Rhythm analyses were performed by AMM using the RAIN and ABSR algorithms.

Timing and spatial scale

Behavioural data: The data analysed in this study were collected from July 27, 2014 to August 31, 2014, during which the camera recorded 2-min video sequences four times a day. This frequency was used because we suspected, based on the literature, the presence of tidal modulation in species behaviour. To detect a cycle, one needs at least 2 observations within this cycle, and 4 is generally recommended. Four observations a day thus allowed for the detection of diurnal tidal signal and possibly semi-diurnal tidal signal while optimising the batteries lifetime for the camera to last a year at the bottom. The spatial scale of the observed mussels was ~1m².

Temporal transcriptomes: for both experiments, data were sampled every 2h 4min for 24h 48min.
In situ experiment: mussels were collected on July 16 and 17, 2017. Laboratory experiment: mussels were collected on July 26, 2017, kept on board for 40h, transferred to the laboratory on July 28, acclimated in the lab for 48h, and sampled on July 30 and 31, 2017.
For each experiment, animals were collected in the same respective areas: their spatial scale were ~1 m² each.
As the periodogram analyses revealed that 12.5 h and 26.3 h were the dominant periods in the behaviour of mussels, the transcriptome analysis was focused on tidal and daily rhythms. We sampled over 24 h 48 min to cover two tidal cycles and a daily cycle. The sampling frequency was chosen to provide a high-resolution transcriptome: the tidal signal requires to sample <every 6h (Nyquist–Shannon sampling theorem). However, the detection of rhythmic transcripts depends on the sampling resolution (see Methods). 2h 04 min was chosen to obtain the highest sampling frequency possible in the deep-sea, with all the constraints related to a ROV and the possibilities to bring samples back to the surface.

Data exclusions

No data were excluded from the analyses.

Reproducibility

Considering the technical constraints related to deep-sea sampling in situ and the maintenance of deep-sea specimen in the laboratory, the experiments could only be performed once.
Despite the technical challenge, all information is provided in the Methods section to allow the experiments to be reproduced.
The number of sequenced animals ensured a high level of robustness for the present work.

Randomization

This is not relevant for our study. For the behavioural analysis, all the mussels that could be identified repeatedly on the pictures were analysed. For the temporal transcriptome in situ : mussels were randomly sampled using the ROV arm. Then 5 to 7 animals were dissected for each time point, all samples complied with quality criteria, and those with the highest quality scores were selected and sent for sequencing. For the temporal transcriptome in the laboratory, all samples complied with quality criteria and all samples were sent for sequencing.

Blinding

Blinding was not relevant for our study. The origin, in situ or laboratory experiment, was clear for each sample.

Did the study involve field work? ☒ Yes ☐ No

Field work, collection and transport

Field conditions

Parameters that were recorded on the field at the time of sampling (temperature and pressure) are provided in Figure 3.
Mussels' habitat was clearly under tidal influence during the sampling period with both temperature and pressure (range 2,550.2-2,551.1 Psi.) oscillating with a ~tidal period (12.3 and 12.7 h ; Dutilleul multi-frequential periodogram analysis, both p-values < 0.05). While the temperature range was small, 4.5-4.7°C, concentrations in sulphides, methane and copper can vary with one order of magnitude between 4.0 and 4.5°C on the LS vent field.

Location

As described above, both vent field are located on the MAR:
Lucky strike vent field: Eiffel tower edifice, N 37° 17.820 W 032° 16.322, 1,688 m depth; pressure and temperature probe, N 37° 17.330 W 032° 16.533, 1,726 m depth.
Menez Gwen vent field: White Flames area, N37° 50.672 W 31° 31.148, 834 m depth.

Access and import/export

The authorization request to work in the Portuguese EEZ during the Momarsat2017 cruise was sent in January 2017. This request presented the scientific objectives of the cruise, and the means to achieve these objectives. The specific sampling program was also described. The authorization to work was sent by the Portuguese Ministerio dos Negocios estrangerios the 26th of June 2017 under the reference : Nota Verbal n°342/2017

Nagoya Protocol on Access and Benefit-sharing (ABS)
An information message was sent (13/01/2017) to Mr Marco Sarmento Rebelo, focal point for Portugal concerning Access and benfit sharing (https://absch.cbd.int/countries/PT).
Mr. Marco Sarmento Rebeo
Departamento de Planeamento e Assuntos Internacionais Instituto da Conservação da Natureza e das Florestas Avenida da

	República, 16 a 16B 1050-191 Lisboa<br>marco.rebelo@icnf.pt
Disturbance	All the work in situ was performed under red light to limit any light contamination.

Reporting for specific materials, systems and methods

We require information from authors about some types of materials, experimental systems and methods used in many studies. Here, indicate whether each material, system or method listed is relevant to your study. If you are not sure if a list item applies to your research, read the appropriate section before selecting a response.

Materials & experimental systems

n/a	Involved in the study
☒	☐ Antibodies
☒	☐ Eukaryotic cell lines
☒	☐ Palaeontology
☐	☒ Animals and other organisms
☒	☐ Human research participants
☒	☐ Clinical data

Methods

n/a	Involved in the study
☒	☐ ChIP-seq
☒	☐ Flow cytometry
☒	☐ MRI-based neuroimaging

Animals and other organisms

Policy information about studies involving animals; ARRIVE guidelines recommended for reporting animal research

Laboratory animals	The study did not involve laboratory animals.
Wild animals	The study was performed on Bathymodiolus azoricus mussels. Mussels were collected in Menez Gwen using the ROV Victor6000, kept on board for 40 h in natural seawater before being transferred to the Lab Horta facility of the Universidade dos Açores.The sex and age could not be determined, but sizes is provided: 63 ± 8 mm shell length (mean ± sd, n=65 animals). Animals were fixed in situ and dissected for sequencing analyses. B. azoricus is a deep-sea species and cannot be released in coastal waters: all animals were dissected to be sacrificed at the end of the experiment.
Field-collected samples	For the laboratory experiment, mussels were acclimated, for 48h only due to technical constraints, in a temperature-controlled room in an aquarium at 6.0 ± 0.5°C and pH 6.8 under a 12h:12h L:D schedule.
Ethics oversight	The experimental procedures comply with French law and with Ifremer institutional guidelines.

Note that full information on the approval of the study protocol must also be provided in the manuscript.
