# Supplementary material for: Biological rhythms in the deep-sea hydrothermal mussel Bathymodiolus azoricus
Source: Nat Commun. 2020 Jul 10;11:3454. doi: 10.1038/s41467-020-17284-4 (PMC7351958; doi:10.1038/s41467-020-17284-4)
Supplement: Supplementary file 4 — Description of Additional Supplementary Files [file 41467_2020_17284_MOESM4_ESM.docx]

**Description of Additional Supplementary Files**

File Name: Supplementary Data 1
Description:
Supplementary Data 1a. List of the most robust rhythmic transcripts in the range 10.4-14.4h for the *in situ* experiment. Normalisation performed with down-sampling and DESeq2. It corresponds to the rhythmic contigs whose period was precisely assessed at 12.4h by either RAIN or ABSR plus those that were detected in the circatidal range by the other algorithm. RAIN and ABSR: two-sided tests. BH and q.value: p-value adjusted for multiple-testing using the Benjamini-Hochberg correction.
Supplementary Data 1b. List of the most robust rhythmic transcripts in the range 20-24.8h for the *in situ* experiment. Normalisation performed with down-sampling and DESeq2. It corresponds to the rhythmic contigs whose period was precisely assessed at 24.8h by either RAIN or ABSR plus those that were detected in the circadian range by the other algorithm. RAIN and ABSR: two-sided tests. BH and q.value: p-value adjusted for multiple-testing using the Benjamini-Hochberg correction.
Supplementary Data 1c. List of the most robust rhythmic transcripts in the range 10.4-14.4h for the laboratory experiment. Normalisation performed with down-sampling and DESeq2. It corresponds to the rhythmic contigs whose period was precisely assessed at 12.4h by either RAIN or ABSR plus those that were detected in the circatidal range by the other algorithm. RAIN and ABSR: two-sided tests. BH and q.value: p-value adjusted for multiple-testing using the Benjamini-Hochberg correction.
Supplementary Data 1d. List of the most robust rhythmic transcripts in the range 20-24.8h for the laboratory experiment. Normalisation performed with down-sampling and DESeq2. It corresponds to the rhythmic contigs whose period was precisely assessed at 24.8h by either RAIN or ABSR plus those that were detected in the circadian range by the other algorithm. RAIN and ABSR: two-sided tests. BH and q.value: p-value adjusted for multiple-testing using the Benjamini-Hochberg correction.

File Name: Supplementary Data 2
Description:
Supplementary Data 2a. Enrichment analysis of all rhythmic transcripts identified in the circatidal range (10,4-14,4h) for the *in situ* experiment.
Supplementary Data 2b. Enrichment analysis of all rhythmic transcripts identified in the circadian range (20-24,8h) for the *in situ* experiment.
Supplementary Data 2c. Enrichment analysis of all rhythmic transcripts identified in the circatidal range (10,4-14,4h) for the laboratory experiment.
Supplementary Data 2d. Enrichment analysis of all rhythmic transcripts identified in the circadian range (20-24,8h) for the laboratory experiment.

File Name: Supplementary Data 3
Description:
Supplementary Data 3a. List of the 152 rhythmic transcripts in the range 10.4-14.4h for both the *in situ* and the laboratory experiments. Normalisation performed with down-sampling and DESeq2. RAIN and ABSR: two-sided tests. BH and q.value: p-value adjusted for multiple-testing using the Benjamini-Hochberg correction.
Supplementary Data 3b. List of the 63 rhythmic transcripts in the range 20-24.8h for both the *in situ* and the laboratory experiments. Normalisation performed with down-sampling and DESeq2.
RAIN and ABSR: two-sided tests. BH and q.value: p-value adjusted for multiple-testing using the Benjamini-Hochberg correction.

File Name: Supplementary Data 4
Description:
Supplementary Data 4a. Reference sequences used to search for putative circadian genes in *Bathymodiolus azoricus*.
Supplementary Data 4b. *Bathymodiolus azoricus* circadian clock proteic sequences use for the phylogenies.
Supplementary Data 4c. Characteristics of *Bathymodiolus azoricus* clock gene nucleotidic sequences.
Supplementary Data 4d. Characteristics of *Bathymodiolus azoricus* clock gene proteic sequences.
Supplementary Data 4e. Sequences used to build the CLOCK/BMAL tree.
Supplementary Data 4f. Sequences used to build the PERIOD tree.
Supplementary Data 4g. Sequences used to build the TIMELESS/TIMEOUT tree.
Supplementary Data 4h. Sequences used to build the CRY/PHOTOLYASES tree.

File Name: Supplementary Movie 1
Description: Switching to red light. The Remotely-Operated Vehicle (ROV) *Victor6000* switched from white to red light prior to approaching the sampling site for the mussel *Bathymodiolus azoricus* on the Eiffel Tower edifice at the Lucky Strike vent field of the Mid-Atlantic Ridge (1,688 m depth).

File Name: Supplementary Movie 2
Description: Sampling procedure for the *in situ* experiment. For each sampling time, 7 to 15 mussels were collected on the Eiffel Tower edifice at the Lucky Strike vent field of the Mid-Atlantic Ridge (1,688 m depth) using the ROV *Victor6000*, intentionally slightly cracked open using the ROV arm, and directly placed in a box filled with in-house RNA stabilising solution to preserve RNA immediately upon sampling.
